# Splicing profile by capture RNA-seq identifies pathogenic germline variants in tumor suppressor genes

**DOI:** 10.1038/s41698-020-0109-y

**Published:** 2020-02-24

**Authors:** Tyler Landrith, Bing Li, Ashley A. Cass, Blair R. Conner, Holly LaDuca, Danielle B. McKenna, Kara N. Maxwell, Susan Domchek, Nichole A. Morman, Christopher Heinlen, Deborah Wham, Cathryn Koptiuch, Jennie Vagher, Ragene Rivera, Ann Bunnell, Gayle Patel, Jennifer L. Geurts, Morgan M. Depas, Shraddha Gaonkar, Sara Pirzadeh-Miller, Rebekah Krukenberg, Meredith Seidel, Robert Pilarski, Meagan Farmer, Khateriaa Pyrtel, Kara Milliron, John Lee, Elizabeth Hoodfar, Deepika Nathan, Amanda C. Ganzak, Sitao Wu, Huy Vuong, Dong Xu, Aarani Arulmoli, Melissa Parra, Lily Hoang, Bhuvan Molparia, Michele Fennessy, Susanne Fox, Sinead Charpentier, Julia Burdette, Tina Pesaran, Jessica Profato, Brandon Smith, Ginger Haynes, Emily Dalton, Joy Rae-Radecki Crandall, Ruth Baxter, Hsiao-Mei Lu, Brigette Tippin-Davis, Aaron Elliott, Elizabeth Chao, Rachid Karam

**Affiliations:** 1 Ambry Genetics, Aliso Viejo, CA USA; 2 University of Pennsylvania, Philadelphia, PA USA; 3 OhioHealth Bing Cancer Center, Columbus, OH USA; 4 Aurora St. Luke’s Medical Center, Milwaukee, WI USA; 5 Huntsman Cancer Institute, Salt Lake City, UT USA; 6 Texas Oncology, El Paso, Fort Worth, and Austin, TX USA; 7 Medical College of Wisconsin, Milwaukee, WI USA; 8 Dana Farber Cancer Institute, Boston, MA USA; 9 University of Texas Southwestern Medical Center, Dallas, TX USA; 10 Community Health Network, Indianapolis, IN USA; 11 Massachusetts General Hospital, Boston, MA USA; 12 Ohio State University Wexner Medical Center and James Comprehensive Cancer Center, Columbus, OH USA; 13 University of Alabama at Birmingham, Birmingham, AL USA; 14 Advocate Health, Chicago, IL USA; 15 University of Michigan, Ann Arbor, MI USA; 16 Cedars-Sinai Medical Center, Los Angeles, CA USA; 17 Kaiser Permanente San Jose Medical Center, San Jose, CA USA; 18 University of California at Irvine, Irvine, CA USA; 19 Smilow Cancer Center, Yale New Haven Health, New Haven, CT USA

**Keywords:** Genetic testing, Next-generation sequencing, Cancer genetics

## Abstract

Germline variants in tumor suppressor genes (TSGs) can result in RNA mis-splicing and predisposition to cancer. However, identification of variants that impact splicing remains a challenge, contributing to a substantial proportion of patients with suspected hereditary cancer syndromes remaining without a molecular diagnosis. To address this, we used capture RNA-sequencing (RNA-seq) to generate a splicing profile of 18 TSGs (*APC*, *ATM*, *BRCA1*, *BRCA2*, *BRIP1*, *CDH1*, *CHEK2*, *MLH1*, *MSH2*, *MSH6*, *MUTYH*, *NF1*, *PALB2*, *PMS2*, *PTEN*, *RAD51C*, *RAD51D*, and *TP53*) in 345 whole-blood samples from healthy donors. We subsequently demonstrated that this approach can detect mis-splicing by comparing splicing profiles from the control dataset to profiles generated from whole blood of individuals previously identified with pathogenic germline splicing variants in these genes. To assess the utility of our TSG splicing profile to prospectively identify pathogenic splicing variants, we performed concurrent capture DNA and RNA-seq in a cohort of 1000 patients with suspected hereditary cancer syndromes. This approach improved the diagnostic yield in this cohort, resulting in a 9.1% relative increase in the detection of pathogenic variants, demonstrating the utility of performing simultaneous DNA and RNA genetic testing in a clinical context.

## Introduction

Splicing is the removal of non-coding sequences (introns) from an RNA molecule followed by the ligation of exons, the protein coding regions of genes.^1,2^ DNA variants can impact this process resulting in RNA mis-splicing, such as skipping of coding sequences or inclusion of non-coding ones into the messenger RNA (mRNA), resulting in potential allele loss-of-function. Aberrant splicing data associated with a DNA variant can be used as evidence of pathogenicity, whereas normal splicing data can be used as evidence of neutrality.^3^ RNA-sequencing (RNA-seq) has shown significant potential for improving the diagnostic yield and resolution of DNA genetic testing, primarily because of the functional splicing data generated by this analysis.^4^ Importantly, RNA-seq also addresses a technical limitation of most clinically available DNA genetic tests, which typically capture only exons and short stretches of the flanking introns. Pathogenic variants (PVs) outside the captured sequence will be missed with a DNA-only approach; however, the addition of RNA-seq provides an opportunity to uncover mis-splicing caused by intronic events, leading to the identification of PVs in the non-coding region of genes.^2^

The first attempts to incorporate RNA-seq into clinical diagnostics have involved whole-transcriptome sequencing (WTS) for patients with rare Mendelian disorders who have remained without a molecular diagnosis despite receiving whole-exome or whole-genome sequencing.^5–8^ The addition of WTS has been shown to increase diagnostic yield by 7–36%, depending on the disease studied. Across all studies, pathogenic splicing variants were identified in regions typically captured by current DNA testing methods as well as deep-intronic regions, highlighting the utility of RNA-seq in both identification and interpretation of disease-causing splicing variants.

Studies have also shown the benefits of RNA-seq for hereditary cancer predisposition genes; however, this approach has been traditionally performed as a follow-up to inconclusive DNA testing. In a recent study, RNA genetic test results facilitated classification of 88% of the cancer gene splicing variants selected for analysis as either pathogenic or benign, and was predicted to impact 1 in 43 individuals if performed simultaneously with DNA testing.^9^ Thus, a substantial proportion of patients currently receiving DNA testing are likely to benefit from the addition of RNA genetic testing. Several studies have also identified pathogenic deep-intronic variants across a range of hereditary cancer conditions, including hereditary breast and ovarian cancer (HBOC),^10,11^ Lynch syndrome,^12,13^ familial adenomatous polyposis,^14^ neurofibromatosis,^15^ and Li-Fraumeni syndrome.^16^ However, the prevalence of cancer-predisposing deep-intronic variants has not been fully explored due to the limited scalability of previous RNA testing methods.

In this study, we test the clinical utility of performing simultaneous capture RNA-seq and DNA multi-gene panel testing (MGPT) analysis on whole blood of 1000 patients receiving genetic testing for hereditary cancer syndromes. The 18 tumor suppressor genes (TSGs) tested were selected because loss of function in these genes have been previously associated with increased cancer risk.^17^ By obtaining RNA splicing data in parallel to DNA, we identified PVs that would have been either missed or classified as inconclusive (variant of unknown significance) with DNA testing alone. To determine whether a given splicing event was aberrant, we built a reference control dataset from 345 healthy blood donors using the same RNA-seq capture approach. This allowed us to generate a normal splicing profile for these TSG against which we compared patient’s splicing profiles. We then confirmed the ability of this approach to detect abnormal splicing by testing 25 positive controls that have been previously identified with a PV known to result in mis-splicing. Finally, we demonstrated that this approach can increase the positive yield of genetic testing through the identification of PVs in these cancer predisposition genes.

## Results

### Splicing profile of TSGs in healthy controls

**Fig. 1:**
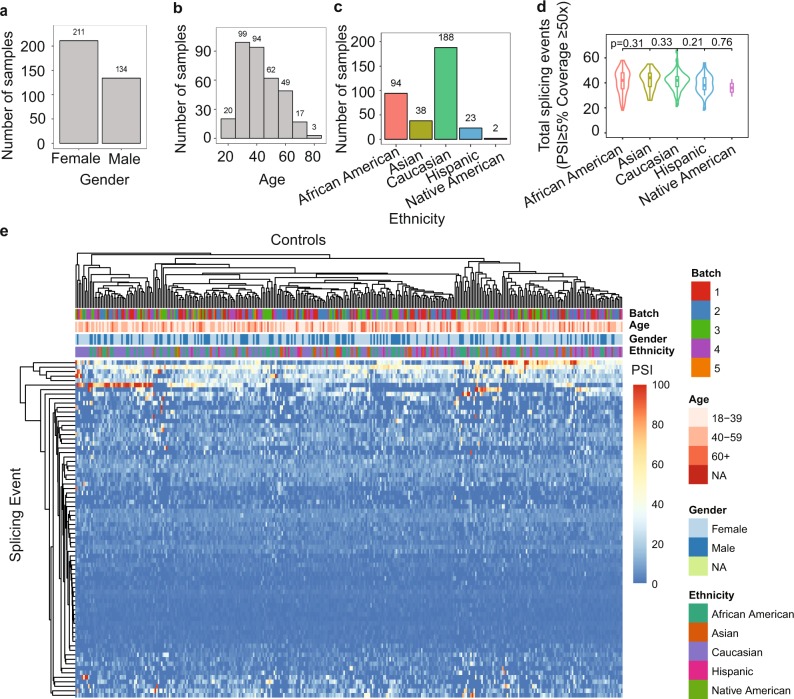
Splicing landscape of 18 TSGs in healthy controls. **a**–**c** Histogram of control samples by ethnicity, age, and gender. **d** Violin plot indicating median number of splicing events detected at ≥5% PSI with coverage ≥50× for each reported ethnicity. *T* test *p* values between neighboring distributions are shown. All other pairwise *t* test *p* values were also non-significant (data not shown). **e** Hierarchical clustering of PSI for splicing events with PSI >1 in at least 150 individuals, assigning PSI to zero if no event was called. Samples (columns) are labeled by batch, age range, gender, and ethnicity.

In this study, a splicing profile of 18 TSGs by capture RNA-seq^18^ was performed in 345 healthy individuals, 25 positive controls, and 1000 patients referred to a clinical diagnostic laboratory for suspicion of hereditary cancer (Supplementary Fig. 1a, b). All genes were found to be expressed in blood (Supplementary Fig. 2a) and had at least 85% of captured exons covered at >50 reads (Supplementary Fig. 2b), our minimum quality thresholds. We compared these expression data to the values found in the GTEx database from whole-blood WTS. All 18 genes were also detected in blood by WTS, although many at lower levels than by capture RNA-seq (Supplementary Table 1). We measured alternative splicing events defined relative to the most clinically relevant isoform for each gene (see Methods and Supplementary Fig. 1c). The relative expression of splicing events was measured by percent splicing index^19^ (PSI) (see Methods). While a minority of alternative splicing events were highly expressed, most exhibited low PSI (Supplementary Fig. 2c). Demographic distribution of the healthy donors is shown in Fig. 1a–c, the majority being Caucasians (54%), females (61%), and 30–40 years old (56%). The number of splicing events was not statistically associated with reported ethnicity (Fig. 1d). Additionally, PSI did not cluster by the observed metadata (age, gender, ethnicity, and batch), suggesting that these factors did not confound the observed PSI variation among healthy donors (Fig. 1e).

**Fig. 2:**
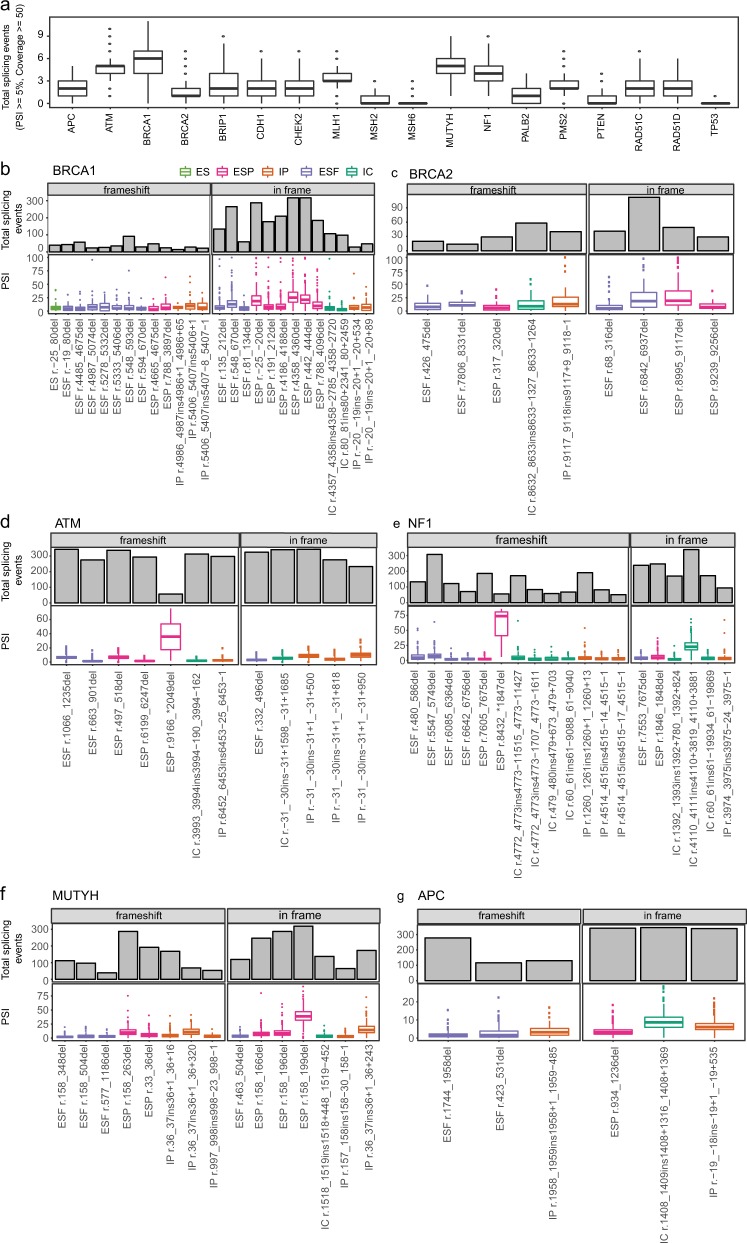
Healthy controls’ alternative splicing events detected in TSG. **a** Boxplot indicating median number of splicing events across controls with ≥5% PSI and coverage ≥50× for each hereditary cancer predisposition gene tested. **b**–**g** Plots indicating frequency (bar graph) and median PSI with interquartile range (boxplot) for alternative splicing events with PSI ≥5 in ≥5% of controls (17/345 controls). For each gene, alternative splicing events are divided into in-frame and frameshift transcripts. Splicing events are as follows: ES = exon skipping—a combination of a full and partial exon skipping event, ESF = full exon skipping—the entire length of the exon is skipped, ESP = partial exon skipping—some portion of the exon is skipped (i.e., alternative acceptor/donor), IC = cryptic exon—an intronic insertion (split reads on 5′ and 3′ end), IP = partial intronic insertion—an extension of the exon into the intron either upstream (5′) or downstream (3′) of the exon. **b**–**d** Hereditary breast and ovarian cancer genes (*BRCA1/BRCA2/ATM*), **e** *NF1*, and **f**, **g** colorectal/polyposis genes’ (*APC*/*MUTYH*) alternative splicing events.

Next, we aimed to characterize alternative splicing events in TSGs common to the healthy population (i.e., PSI ≥5% in ≥5% of healthy donors). Different genes appeared to tolerate different degrees of alternative splicing. *ATM*, *BRCA1*, *MUTYH*, and *NF1* displayed the highest median number of splicing events and *MSH2*, *MSH6*, *PTEN*, and *TP53* the lowest (Fig. 2a, Supplementary Figs. 3–5). Interestingly, the two genes with the highest number of exons also had a high median splice event count (*ATM*, *NF1*). In order to determine if the observed data might be explained by the number of exons, we calculated the Pearson’s correlation coefficient between median splice event count and exon number for all 18 genes. Overall, we observed that there is a correlation between number of exons and median splice event count (*ρ* = 0.57, *p* = 0.01). While 26 recurrent splicing events were identified in *BRCA1* (Fig. 2b), only nine such events were observed in *BRCA2* (Fig. 2c). In *ATM*, 12 events were identified, eight of which were detected in most of the healthy donors at low median PSI (Fig. 2d). Some alternative splicing events detected represent known alternative isoforms. For example, *NF1* r.4110_4111ins4110 + 3819_4110 + 3881 corresponds to inclusion of an exon annotated in isoform NM_001042492,^20,21^ and exons specific to NM_001354896 and NM_001354900^22,23^ were detected in *APC* in all controls at low levels (Fig. 2e, g). In *MUTYH*, 15 events were identified, most with median PSI <20% (Fig. 2f). Interestingly, 100% of controls expressed an in-frame partial skipping of exon 3 in *NF1* (r.158_199del, median PSI = 38.69; interquartile range = 31.91–47.13) (Fig. 2f). Four partial exon 3 skipping events were detected in *MUTYH*, consistent with previous reports.^24–26^ Altogether, these data provide a reliable splicing landscape for these TSGs in whole blood of healthy individuals, against which putative abnormal splicing events observed in patients can be analyzed.

### Splicing profile helps detect mis-splicing

**Fig. 3:**
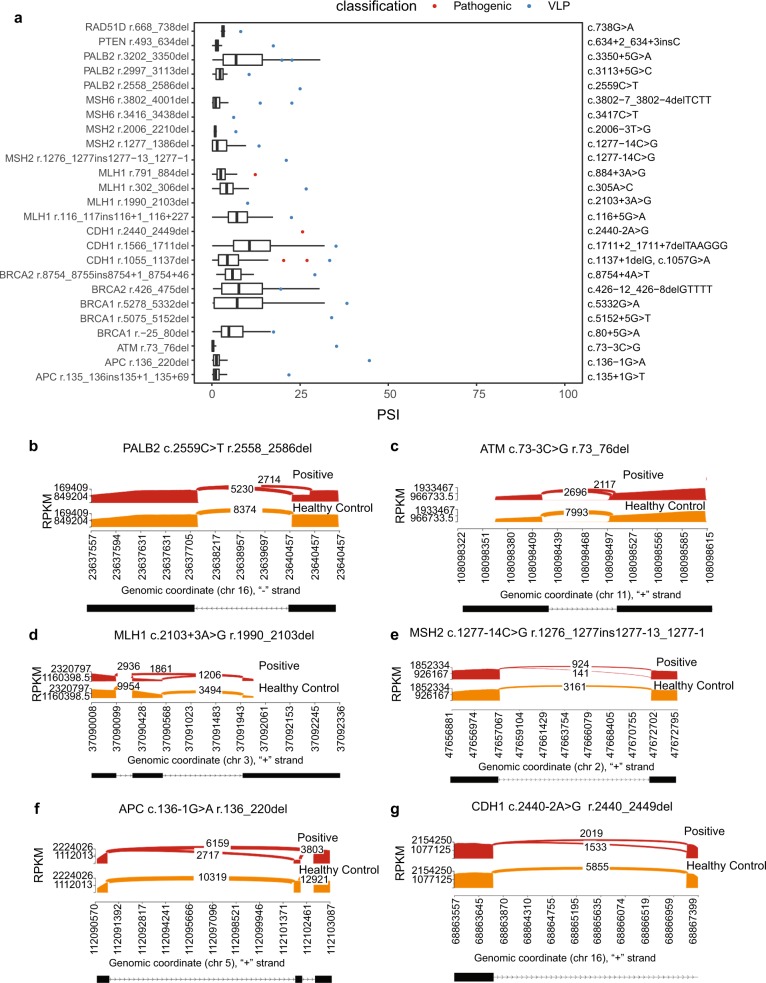
Splicing profiling can detect mis-splicing in individuals carrying germline likely pathogenic/pathogenic variants. **a** Plot comparing PSI of alternative splicing event associated with the given pathogenic (red) or variant likely pathogenic (VLP; blue) germline DNA variant obtained from proband against median PSI and interquartile range for that splicing event in controls, excluding samples in which the splicing event was not detected (black boxplot; outlier points are not shown). If the event was not found in controls, only the proband PSI was plotted. All PSI values were significantly higher than the mean control PSI (one-sample two-sided *t* test, *p* < 1 × 10^−55^). For CDH1 r.1055_1137del, the PSI values of 20 and 27 were observed in individuals with the pathogenic variant c.1137 + 1delG and the PSI value of 33 was observed in an individual with the VLP c.1057G > A. **b**–**g** Sashimi plot indicating reads supporting alternative splicing obtained via CloneSeq for HBOC (**b**, **c**), HNPCC (**d**, **e**), and HDGC (hereditary diffuse gastric cancer)/FAP (**f**, **g**) variants. **b** Partial skipping of CDS6 in *PALB2*. **c** Partial skipping of CDS2 in *ATM*. **d** Full skipping of CDS18 in *MLH1*. **e** Partial retention of intron 7 in *MSH2*. **f** Full skipping of CDS2 in *APC*. **g** Partial skipping of CDS16 in *CDH1*.

Having generated a splicing reference dataset to contextualize putative pathogenic splicing events, we tested the 25 positive controls. These were blood samples from individuals heterozygous for likely PVs/PVs known to affect splicing of genes associated with HBOC, colorectal cancer predisposition (e.g., Lynch syndrome, familial adenomatous polyposis), or hereditary diffuse gastric cancer. For all tested variants, the PSI for the variant-associated splicing event was greater than the mean PSI among controls (one-sample two-sided *t* test, *p* < 1 × 10^−55^) (Fig. 3a and Supplementary Table 2). These results were validated using a second orthogonal methodology, CloneSeq, a highly sensitive and specific targeted RNA-seq assay based on cloning of reverse transcription-PCR (RT-PCR) products, followed by massively parallel sequencing of the cloned transcripts.^27^ CloneSeq results are displayed as Sashimi plots (Fig. 3b–g and Supplementary Figs. 6–8), which provide an absolute number of aligned reads that enables comparison of exon usage across probands and controls.^28^ Among the positive controls, we observed concordant results between the CloneSeq and capture RNA-seq for all clinically significant splicing events (Fig. 3b–g and Supplementary Figs. 6–8), demonstrating the utility of capture RNA-seq to detect mis-splicing associated with germline PVs.

### Paired DNA and RNA genetic testing identifies PVs in TSG

Finally, we sought to assess in a clinical context the ability of whole-blood splicing profile by capture RNA-seq to detect variants that result in mis-splicing of these 18 TSGs. We performed simultaneous capture RNA-seq and DNA MGPT in 1000 consecutive patients referred for clinical inherited cancer predisposition testing, to evaluate the change in the diagnostic yield upon adding concurrent RNA testing to MGPT. RNA data was incorporated during variant assessment as recommended by the ACMG/AMP expert guidelines,^3^ alongside with other available lines of evidence.^29,30^ In total, 84 individuals received positive results (PVs/likely PVs) after performing consecutive DNA-sequencing and RNA-seq, versus 77 individuals who would have received positive results if MGPT was performed alone, resulting in a 9.1% relative increase in the diagnostic rate. All abnormal splicing events were confirmed by Sanger sequencing of cloned RT-PCR products (Supplementary Fig. 9).

**Fig. 4:**
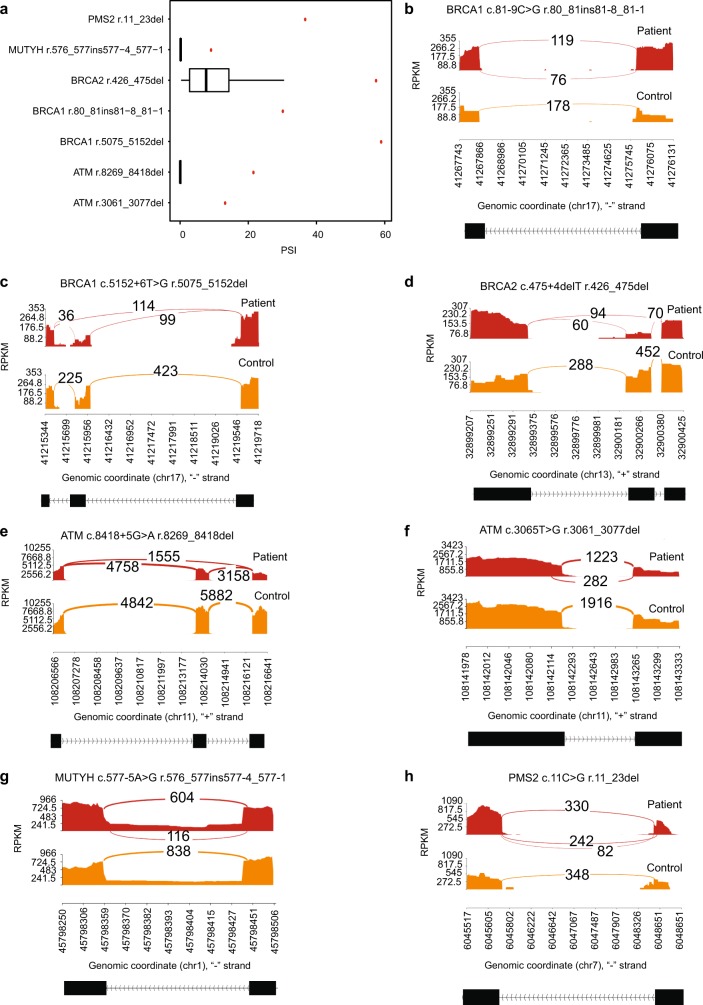
Pathogenic variants identified in a consecutive cohort of 1000 patients analyzed by paired DNA and RNA genetic testing. All PSI values shown were significantly higher than the mean control PSI (one-sample two-sided *t* test, *p* < 1 × 10^−55^). **a** PSI for variant-associated r dot, shown in red, compared to boxplot representing median and interquartile range of PSI for healthy controls, if applicable (outlier points are not shown). **b** Sashimi plot of *BRCA1* r. r.80_81ins81-8_81-1 indicating coverage and junction reads supporting the 8 bp partial intronic insertion. Patient plot is tuna-colored, whereas control plot is salmon colored. **c** Sashimi plot of *BRCA1* r.5075_5152del indicating coverage and junction reads supporting the coding exon 16 skipping. **d** Sashimi plot of *BRCA2* r.426_475del indicating coverage and junction reads supporting the coding exon 4 skipping. **e** Sashimi plot of *ATM* r.8269_8418del indicating coverage and junction reads supporting the coding exon 56 skipping. **f** Sashimi plot of *ATM* r.3061_3077del indicating coverage and junction reads supporting the partial skipping of coding exon 19. **g** Sashimi plot of *MUTYH* r.576_577ins577-4_577-1 indicating coverage and junction reads supporting the partial intron 7 retention. **h** Sashimi plot of *PMS2* r.11_23del indicating coverage and junction reads supporting the partial skipping of coding exon 1.

Two cases exemplify the utility of splicing profile by capture RNA-seq in identifying PVs in the HBOC predisposition gene *BRCA1*. In the first case, the proband is a female with Caucasian/Asian descent diagnosed with breast cancer at the age of 25 and 33 years, and high-grade papillary serous ovarian cancer at the age of 44 years. She had multiple HBOC tests over the last 18 years which were inconclusive. Splicing profile identified an out-of-frame partial retention of intron 2 (r.80_81ins81-8_81-1), which was not detected in controls (Fig. 4a, b). Analysis of the DNA identified a cytosine to guanine substitution nine nucleotides upstream from *BRCA1* intron 2 (c.81 − 9C > G), which is predicted in silico^31,32^ to weaken the native splice acceptor and create a cryptic acceptor, as confirmed by the RNA data. The resulting aberrant transcript is predicted to cause a glutamic acid to leucine substitution at position 29 in the amino acid chain and result in a frameshift causing a premature termination codon (p.E29Lfs*5). In the second case, the proband is a female Caucasian of Ashkenazi Jewish descent diagnosed with breast cancer at the age of 49 years. Skipping of exon 17 (r.5075_5152del) was observed in the proband but not in controls (Fig. 4a, c). A *BRCA1* variant six nucleotides downstream of exon 17 was detected by DNA MGPT (c.5152 + 6T > G), which is predicted to abolish the splice donor. This in turn is predicted to result in the deletion of amino acids at positions 1692 through 1718 and the insertion of a glycine (p.D1692_W1718delinsG). Saturation mutagenesis studies in *BRCA1*^30^ support that such a change would result in a non-functional protein.

In *BRCA2*, full skipping of exon 5 (r. 426_475del) was observed at approximately twice the highest PSI observed in controls (57.7%) (Fig. 4a, d) in a Caucasian female diagnosed with breast cancer at the age of 53 years. This was associated with a rare, germline thymine deletion four nucleotides downstream from the exon (c.475 + 4delT), predicted to abolish the native donor splice site. The aberrant transcript is predicted to result in a proline to glycine substitution at position 143 and a frameshift resulting in a premature termination codon (p.P143Gfs*23). This variant was also previously detected in our laboratory cohort in a Caucasian male diagnosed with breast cancer at the age of 81 years, and prostate cancer at the age of 69 years.

In *ATM*, two splicing variants were reclassified from inconclusive to positive due to RNA evidence. The first variant was detected in a Hispanic female proband diagnosed with breast cancer at the age of 64 years. The *ATM* variant is a rare guanine to adenine substitution five nucleotides downstream from exon 56 (c.8418 + 5G > A). The splicing in silico tools predicted a weakened donor splice site. The aberrant transcript identified by capture RNA-seq (Fig. 4a, e) is predicted to result in amino acid deletions at positions 2757 through 2806, which is located in a critical function domain for the protein (p.V2757_M2806del). In another *ATM* case (Fig. 4a, f) we identified a partial skipping of exon 19 (r.3061_3077del) in a Caucasian female diagnosed with invasive ductal carcinoma of the breast at the age of 33 years. This was associated with a thymine to guanine substitution located within the same exon (c.3065T > G). The RNA data confirmed splicing predictions that the alteration would strengthen a cryptic donor splice site. The transcript is predicted to result in a valine to alanine substitution at position 1021 with a frameshift and resulting in a premature termination codon.

In *MUTYH* (Fig. 4a, g) we detected a partial intron 7 retention (r.576_577ins577-4_577-1) in a 31-year-old female with no personal history of cancer. This was associated with an adenine to guanine substitution five nucleotide upstream from exon 8 (c.577 − 5A > G). The transcript was predicted to result in valine to serine substitution at position 193 with a frameshift and premature termination codon. *MUTYH* causes an autosomal recessive polyposis syndrome, *MUTYH*-associated polyposis. In accordance with the expected phenotype, this variant has been previously identified in our laboratory cohort to co-occur with the founder PV *MUTYH* p.G396D in two patients with severe polyposis.

Finally, we detected in a Caucasian female diagnosed with breast cancer at the age of 65 years partial skipping of *PMS2* exon 1 (r. 11_23del), which was absent from controls (Fig. 4a, h). This splicing event was associated with a rare germline exonic variant in *PMS2* (c.11C > G). Predictions in silico indicated that this cytosine to guanine substitution results in the creation of a novel donor site that is 13 base pairs upstream from the native splice donor site, and this was confirmed by the RNA data. The aberrant transcript is predicted to result in an alanine to valine substitution as position 4 with a frameshift and premature termination codon (p.A4VFS*26).

## Discussion

Results from our study demonstrate the feasibility and utility of capture RNA-seq in identifying patients with clinically actionable variants that would have been either missed with DNA testing alone or classified as inconclusive. This whole-blood assay also offers a clinical alternative to other less scalable methods for analysis of splicing variants, such as minigene assays.^33–38^ In this cohort, the 9.1% relative increase in the diagnostic yield for high-risk cancer genes is comparable if not superior to the relative increase in yield gained by the introduction of DNA copy number variation analysis.^17^ We anticipate that RNA-seq will have an even higher impact in the diagnostic yield among specific genes and phenotypic sub-groups, which will be evaluated as the testing cohort continues to expand.

The additional increase in diagnostic yield offered by RNA-seq improves a clinician’s ability to accurately apply personalized management strategies for early detection, cancer risk reduction, and treatment. In six of the seven cases with RNA-seq-related positive test results, substantial changes to medical management would be recommended based on current guidelines—not only for the probands but also for family members who test positive as well. PVs in *BRCA1* and *BRCA2* have implications not only for early detection and risk reduction of several associated cancer types but also have the potential to inform eligibility for PARP inhibitor therapy.^39^ Similarly, early detection and prevention options are recommended for individuals with *PMS2* PVs.^40^ For individuals with pathogenic *ATM* variants, increased breast cancer surveillance is recommended.^39^

Our capture RNA-seq approach required the generation of a unique control dataset to characterize the splicing landscape of TSGs in healthy individuals. Evaluating whether alternative splicing events are part of normal biological variation is an essential quality component of a clinical grade RNA-seq assay. In the absence of control data, laboratories risk over-interpreting splicing events as pathogenic. Considering the frequency of alternative splicing events in the healthy control population reported this study, failure to consider the natural splicing landscape in cancer genes could result in misclassifications.

Previous studies have leveraged WTS approaches to assess for pathogenic splicing variants across the genome. While our capture RNA-seq approach is more limited in the number of genes analyzed, it has a distinct advantage over WTS as it offers increased depth of coverage for genes that are not highly expressed in blood yet have a known clinical impact (e.g., *BRCA1* and *BRCA2*). Along with further development of standards for the interpretation of RNA findings and the generation of publicly available databases for alternative splicing events, this approach should also be considered to accompany DNA testing panels for other hereditary diseases.

In summary, our results demonstrate the clinical utility of adding capture RNA-seq to routine genetic testing. The splicing profile approach we described can obtain a high-resolution picture of abnormal splicing events for clinically relevant genes, consequently improving the positive yield of genetic testing and supporting its adoption in a clinical context.

## Methods

### Ethics

This study was approved and carried out in accordance with the recommendations of the Western Institutional Review Board (Puyallup, Washington). All participants provided written informed consent to take part in the study.

### Samples

For the healthy controls and retrospective cohort, peripheral blood was drawn from 345 healthy donors and 83 patients participating in the Ambry Genetics Family Studies program, respectively. For the prospective cohort, patients were referred for paired DNA and RNA MGPT to Ambry Genetics by their physician or healthcare provider from 17 medical centers across the United States as part of routine clinical genetic cancer risk assessment (*n* = 1000). Harvested blood was collected and stored in PAXgene™ Blood RNA tubes (PreAnalytiX). For the retrospective cohort, RNA was extracted using either a manual method (PAXgene™ Blood RNA Kit—PreAnalytiX) or an automated one (Thermo MagMAX™ for Stabilized Blood Tubes RNA Isolation Kit, compatible with PAXgene™ Blood RNA Tubes on Thermo King Fisher Presto). RNA yield and RNA integrity number were quantified using a TapeStation with RNA ScreenTape (Agilent).

### DNA-sequencing and RNA-seq

For capture RNA-seq, complementary DNA (cDNA) libraries were generated using a RiboErase + RNA Hyper Prep Kit (KAPA Biosystems) as previously described.^41^ Briefly, ribosomal RNA was captured via DNA oligonucleotide probes and digested using RNAse H. The enriched RNA was heat fragmented at 94 °C for 6 min and first-strand cDNA was synthesized immediately thereafter. Second-strand synthesis and A-tailing was followed by adapter ligation, SPRI cleanup, and index PCR. The amplified and SPRI cleaned libraries were then assessed for yield and aberrant peaks using a Tapestation (Agilent) with D1000 ScreenTape. Libraries passing the quality assessment were pooled and subjected to bait capture as described in Mercer et al.^18^ The post-capture libraries were subjected to a second round of index PCR amplification, SPRI cleaned, and then assessed with the Tapestation. Twelve library pools (96 samples) were sequenced at 2 × 150 bp on a NextSeq 500 (Illumina). CloneSeq and Sanger sequencing of individual colonies was performed as previously described.^27^ DNA MGPT was performed as previously described.^42^

### Sequencing data processing

Sequencing raw data processing was described previously.^43^ De-multiplexing was done with the RTA software 1.17.21.3 (Real Time Analysis, Illumina Inc., San Diego, CA, USA) and bcl2fastq Conversion software v1.8 (Illumina Inc., San Diego, CA, USA). RNA samples that passed sequencing quality control (QC) criteria (percentage of Q30 bases >75%, mean base quality >30, and percentage of perfect index >85%) were used for downstream analysis. Paired-end RNA-seq reads (2 × 150 bp) were first aligned to the hg19 human reference genome using the STAR aligner v2.6.0c. Average coverage for each exon from the 18 genes (Supplementary Table 3) was calculated. A sample passed sequencing coverage QC cutoff if ≥85% of exons from the 18 genes have average coverage ≥50×. RNA samples that failed the coverage or QC cutoffs were re-prepared and re-sequenced. Data from samples that again failed the covarage and/or QC cutoffs are not included in the study. Mapped reads were then analyzed by custom in-house software based on Schafer et al.^19^ to detect splicing events relative to canonical RefSeq transcripts for in the 18 hereditary cancer predisposition genes (Supplementary Fig. 2a and Supplementary Table 3). PMS2 exons 11 to 15 were excluded from analysis due to interference with >99% homologous pseudogene PMS2CL. One reference isoform was chosen for each gene. Relative to this isoform, the PSI value was defined as the number of reads supporting the alternative splicing event (i.e., the non-canonical/abnormal event) divided by the number of all reads in the region covering splicing event. This calculation differs slightly from previously published methods of splicing quantification in that it considers abnormal splicing reads as the numerator as opposed to the number of inclusion reads.^19^ To test for differential PSI between the patient and healthy controls, we used a one-sample two-sided *t* test. The Holm–Sidak correction was used to adjust the *p* values for multiple hypothesis testing. A splicing event from a patient was considered significantly higher than healthy controls if its PSI was ≥5% and larger than the mean PSI of healthy donors, the adjusted *p* value was <0.05, and the number of all reads in the region covering splicing event was ≥50. Splicing events with lower PSI than controls were not considered.

Bar plots, box plots, and violin plots were generated using ggplot2 package (v3.5.3) from R v3.5.1 with default settings. Hierarchical clustering was generated using pheatmap v3.5.2. Sashimi plots were generated using rmats2sashimiplot (https://github.com/Xinglab/rmats2sashimiplot).

### Interpretation of genetic test results

Classification of germline variants identified by MGPT was performed following the American College of Medical Genetics and Genomics and the Association for Molecular Pathology (ACMG/AMP) guidelines.^3,44,45^ Multiple lines of evidence are combined, to reach one of the following classifications: PV; variant, likely pathogenic; variant of unknown significance; variant, likely benign; or benign variant. ACMG/AMP guidelines place greater weight on experimental evidence of abnormal splicing compared with in silico predictors. In this study, RNA results were used as weighted evidence towards pathogenic or benign classification in the assessment of identified germline variants, following the ACMG/AMP guideline recommendations. Updated variant classifications are publicly available at ClinVar (https://www.ncbi.nlm.nih.gov/clinvar/).

### Diagnostic rate

Among the 1000 patients referred to Ambry for suspicion of hereditary cancer, patients with a PV within any of the 18 TSGs prior to the incorporation of RNA data were considered the denominator for diagnostic rate (*n* = 77). This calculation excluded moderate risk mutations (MUTYH carriers, CHEK2 I57T and APC I1307K). Physicians/healthcare providers could select up to 18 of the TSGs on the RNA panel for testing (all 18 genes were not selected in every case). The numerator for diagnostic rate was the difference between the number of patients with a PV after incorporating RNA data (*n* = 84) and the number of patients with a PV prior to RNA data (*n* = 77). This yielded a relative percent increase of 9.1%.

### Reporting summary

Further information on research design is available in the Nature Research Reporting Summary linked to this article.

## Supplementary information

Supplementary Material

Reporting Summary

## Data Availability

RNA sequence data that support the findings of this study have been deposited in NCBI Sequence Read Archive (SRA) with the submission accession number PRJNA603342. The patient data that support the findings of this study are available on request from the corresponding author (R.K.). The patient data are not publicly available due to them containing information that could compromise research participant privacy/consent.
