# Supplementary material for: Splicing profile by capture RNA-seq identifies pathogenic germline variants in tumor suppressor genes
Source: NPJ Precis Oncol. 2020 Feb 24;4:4. doi: 10.1038/s41698-020-0109-y (PMC7039900; doi:10.1038/s41698-020-0109-y)
Supplement: Supplementary file 1 — Supplementary Material [file 41698_2020_109_MOESM1_ESM.pdf]

Supplementary Materials

Splicing profile by capture RNA-seq identifies pathogenic germline variants in tumor suppressor genes

Tyler Landrith[1]*, Bing Li[1]*, Ashley Cass[1], Blair Conner[1], Holly LaDuca[1], Danielle McKenna[2], Kara N. Maxwell[2], Susan Domchek[2], Nichole A. Morman[3], Christopher Heinlen[3], Deborah Wham[4], Cathryn Koptiuch[5], Jennie Vagher[5], Ragene Rivera[6], Ann Bunnell[6], Gayle Patel[6], Jennifer L. Geurts[7], Morgan M. Depas[7], Shraddha Gaonkar[8], Sara Pirzadeh-Miller[9], Rebekah Krukenberg[10], Meredith Seidel[11], Robert Pilarski[12], Meagan Farmer[13], Khateriaa Pyrtel[14], Kara Milliron[15], John Lee[16], Elizabeth Hoodfar[17], Deepika Nathan[18], Amanda C. Ganzak[19], Sitao Wu[1], Huy Vuong[1], Dong Xu[1], Aarani Arulmoli[1], Melissa Parra[1], Lily Hoang[1], Bhuvan Molparia[1], Michele Fennessy[1], Susanne Fox[1], Sinead Charpentier[1], Julia Burdette[1], Tina Pesaran[1], Jessica Profato[1], Brandon Smith[1], Ginger Haynes[1], Emily Dalton[1], Joy Rae-Radecki Crandall[1], Ruth Baxter[1], Hsiao-Mei Lu[1], Brigette Tippin-Davis[1], Aaron Elliott[1], Elizabeth Chao[1,18], Rachid Karam[1#]

[1]Ambry Genetics, Aliso Viejo, CA. [2]University of Pennsylvania, Philadelphia, PA. [3]OhioHealth Bing Cancer Center, Columbus, OH. [4]Aurora St. Luke's Medical Center, Milwaukee, WI. [5]Huntsman Cancer Institute, Salt Lake City, UT. [6]Texas Oncology, Austin and El Paso, TX. [7]Medical College of Wisconsin, Milwaukee, WI. [8] Dana Farber Cancer Institute, Boston, MA. [9] University of Texas Southwestern Medical Center, Dallas, TX. [10]Community Health Network, Indianapolis, IN. [11]Massachusetts General Hospital, Boston, MA. [12] Ohio State University Wexner Medical Center and James Comprehensive Cancer Center, Columbus, OH. [13]University of Alabama at Birmingham, Birmingham, AL. [14]Advocate Health, Chicago, IL. [15]University of Michigan, Ann Arbor, MI. [16]Cedars-Sinai Medical Center, Los Angeles, CA. [17]Kaiser Permanente San Jose Medical Center, San Jose, CA. [18]University of California at Irvine, Irvine, CA. [19] Smilow Cancer Center, Yale New Haven Health, New Haven, CT.

*Both authors contributed equally to work.

[#]Corresponding author:

Rachid Karam, MD, PhD

15 Argonaut, Aliso Viejo, CA 92656

Tel: (949) 457-4347

Email: rkaram@ambrygen.com

Supplementary Figure 1

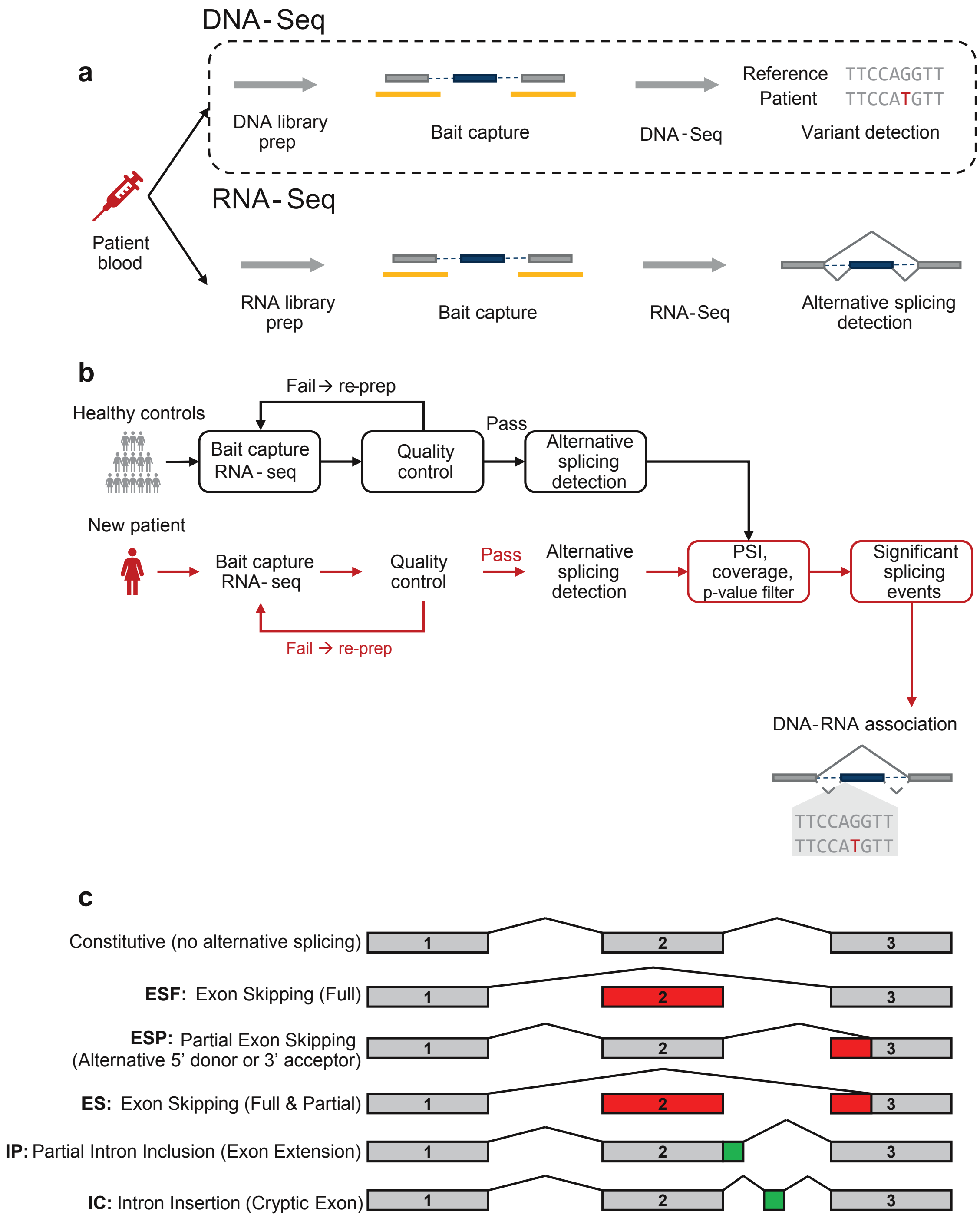

Supplementary Figure 1: Laboratory and analysis workflow. a) Total DNA is extracted from patient blood and libraries are prepared, probes targeting exons of interest are used for bait capture. The enriched libraries are sequenced and the results are analyzed against a reference sequence to detect variants. In parallel, total RNA is extracted from patient blood and cDNA libraries are prepared. Exon targeting probes are used for bait capture to allow detection of novel exons and the enriched libraries are sequenced and results processed with a custom analysis pipeline. b) Healthy controls and patient samples are analyzed identically. Once samples have passed pipeline QC (85% of exons with coverage >50x), splicing analysis is performed, and coverage and PSI cutoffs are applied (PSI>5% with splicing event coverage>50x). Only PSI values that are significantly higher than the mean control PSI are reported. Alternative splicing events are then associated with a given DNA splicing variant, here an exon skipping event associated with the abolishment of the native acceptor site is shown. c) Splicing events reported in this analysis include full exon skipping, partial exon skipping (alternative 5' donor or 3' acceptor), full and partial exon skipping, partial intron retention (exon extension), and intron inclusion (cryptic exon) relative to the canonical transcript with no alternative splicing.

Supplementary Figure 2

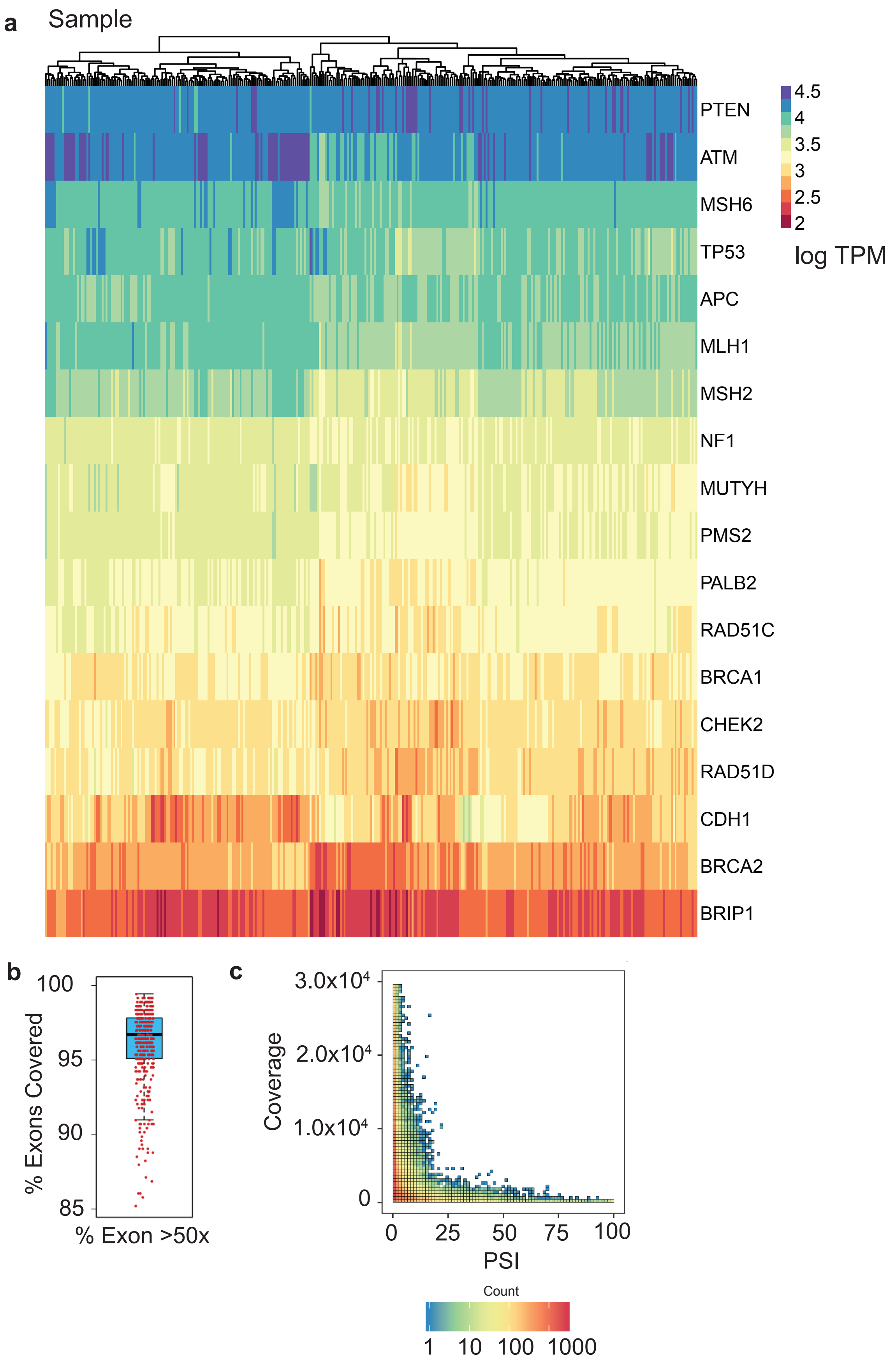

Supplementary Figure 2: Global profile of healthy control dataset. a) Expression heatmap of TSGs displayed as transcripts per million reads (TPM) for all healthy controls. b) Percentage of exons with >50x coverage for each healthy control. All samples had ≥85% exons with >50x coverage. c) Two-dimensional histogram of PSI for all splicing events detected among controls plotted against coverage.

Supplementary Figure 3

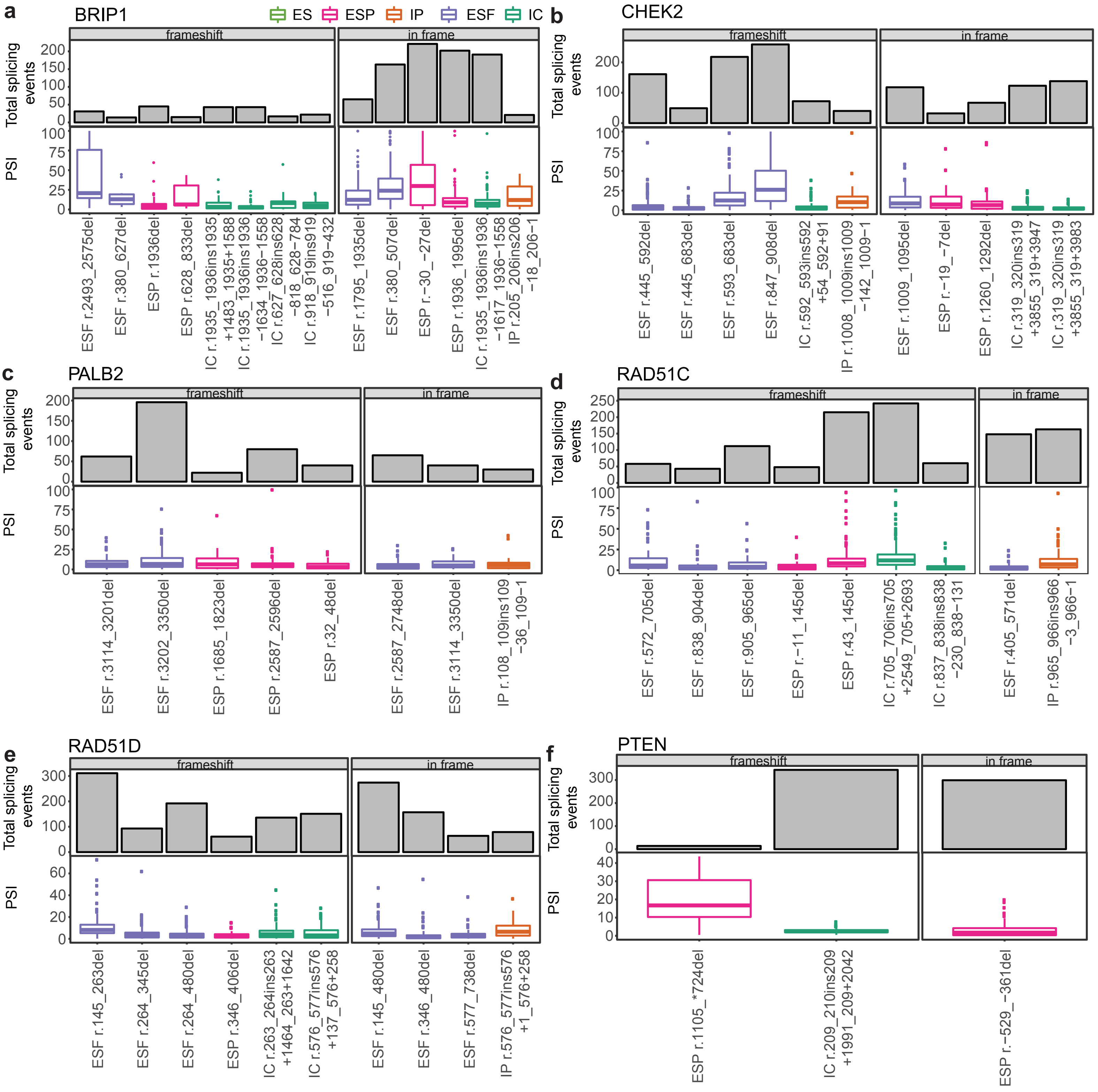

Supplementary Figure 4: Healthy controls' alternative splicing events detected in HBOC genes. Each boxplot indicates median PSI with interquartile range for alternative splicing events with PSI≥5 in ≥5% of controls. For each gene, alternative splicing events are divided into in-frame and out-of-frame transcripts. Splicing events are as follows: ES=Exon skipping-a combination of a full and partial exon skipping event, ESF=Full exon skipping-the entire length of the exon is skipped, ESP=Partial exon skipping-some portion of the exon is skipped (i.e. alternative acceptor/donor), IC=Cryptic exon-an intronic insertion (split reads on 5' and 3' end), IP=Partial intronic insertion-an extension of the exon into the intron either upstream (5') or downstream (3') of the exon. a) *BRIP1* b) *CHEK2* c) *PALB2* d) *PTEN* e) *RAD51C* f) *RAD51D*

Supplementary Figure 4

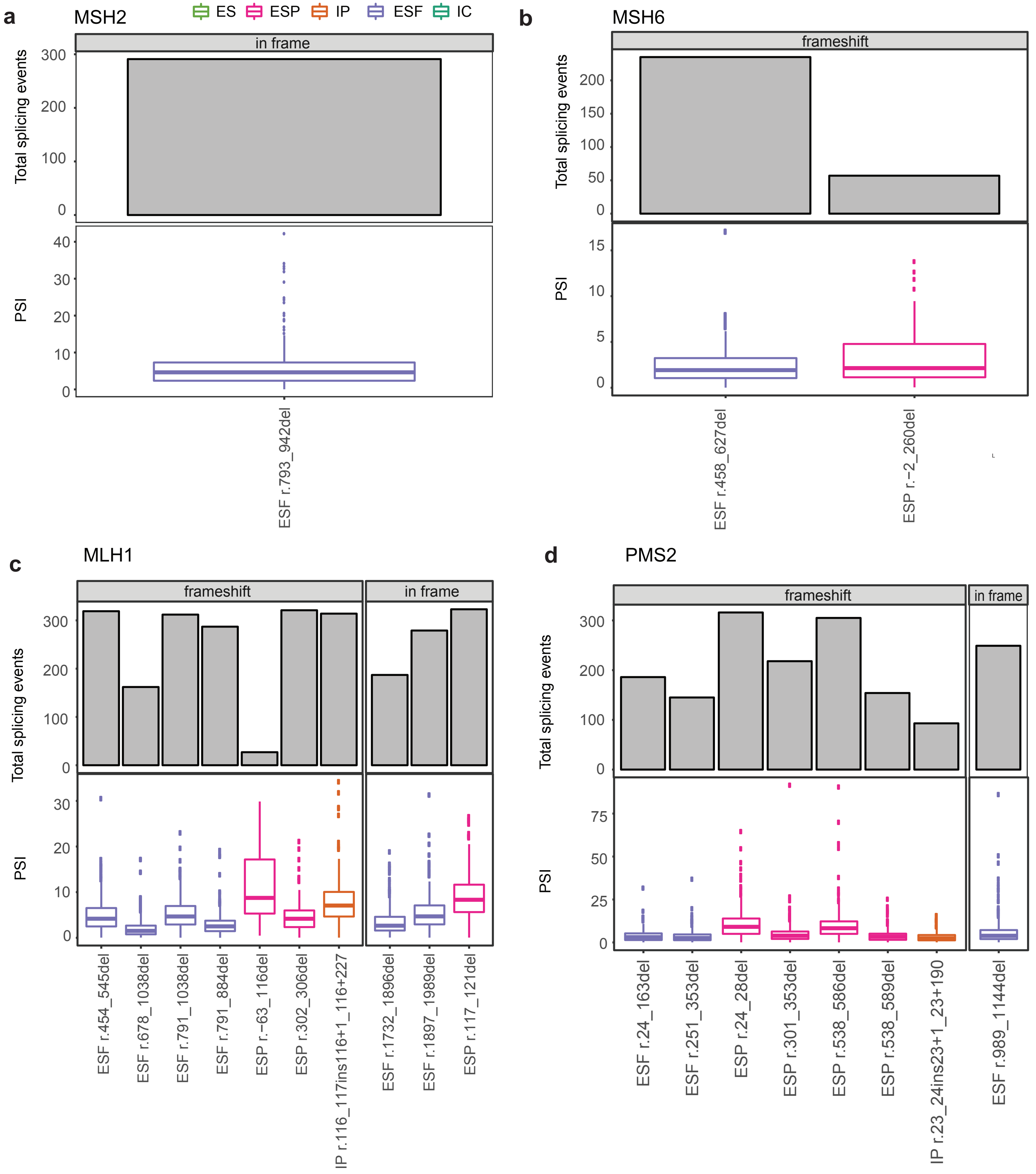

Supplementary Figure 4: Healthy controls' alternative splicing events detected in HNPCC genes. Each boxplot indicates median PSI with interquartile range for alternative splicing events with PSI≥5 in ≥5% of controls. For each gene, alternative splicing events are divided into in-frame and out-of-frame transcripts. Splicing events are as follows: ES=Exon skipping-a combination of a full and partial exon skipping event, ESF=Full exon skipping-the entire length of the exon is skipped, ESP=Partial exon skipping-some portion of the exon is skipped (i.e. alternative acceptor/donor), IC=Cryptic exon-an intronic insertion (split reads on 5' and 3' end), IP=Partial intronic insertion-an extension of the exon into the intron either upstream (5') or downstream (3') of the exon. a) *MSH2* b) *MSH6* c) *MLH1* d) *PMS2*

Supplementary Figure 5

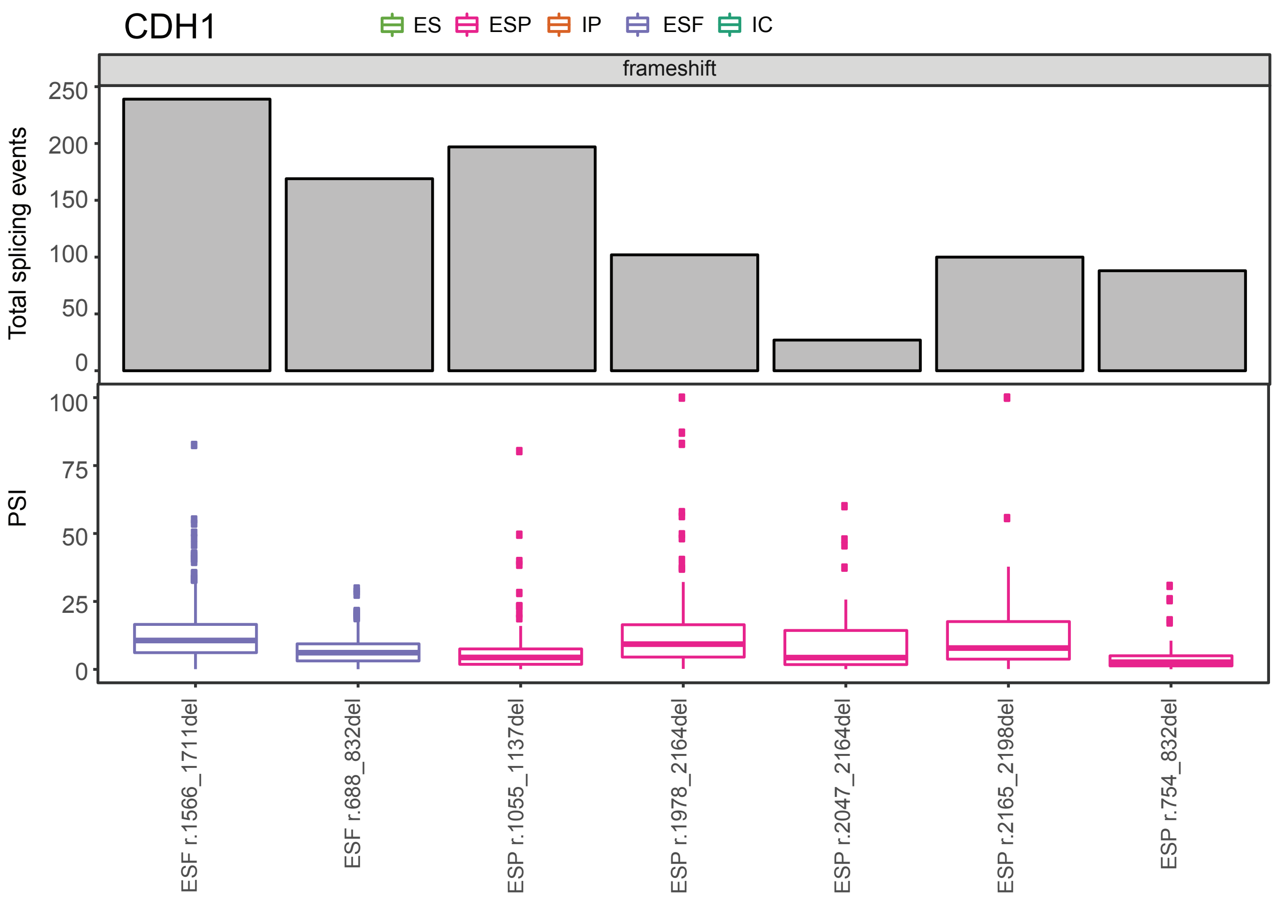

Supplementary Figure 5: Healthy controls' alternative splicing events detected in *CDH1*. Each boxplot indicates median PSI with interquartile range for alternative splicing events with PSI≥5 in ≥5% of controls. For each gene, alternative splicing events are divided into in-frame and out-of-frame transcripts. Splicing events are as follows: ES=Exon skipping-a combination of a full and partial exon skipping event, ESF=Full exon skipping-the entire length of the exon is skipped, ESP=Partial exon skipping-some portion of the exon is skipped (i.e. alternative acceptor/donor), IC=Cryptic exon-an intronic insertion (split reads on 5' and 3' end), IP=Partial intronic insertion-an extension of the exon into the intron either upstream (5') or downstream (3') of the exon.

Supplementary Figure 6

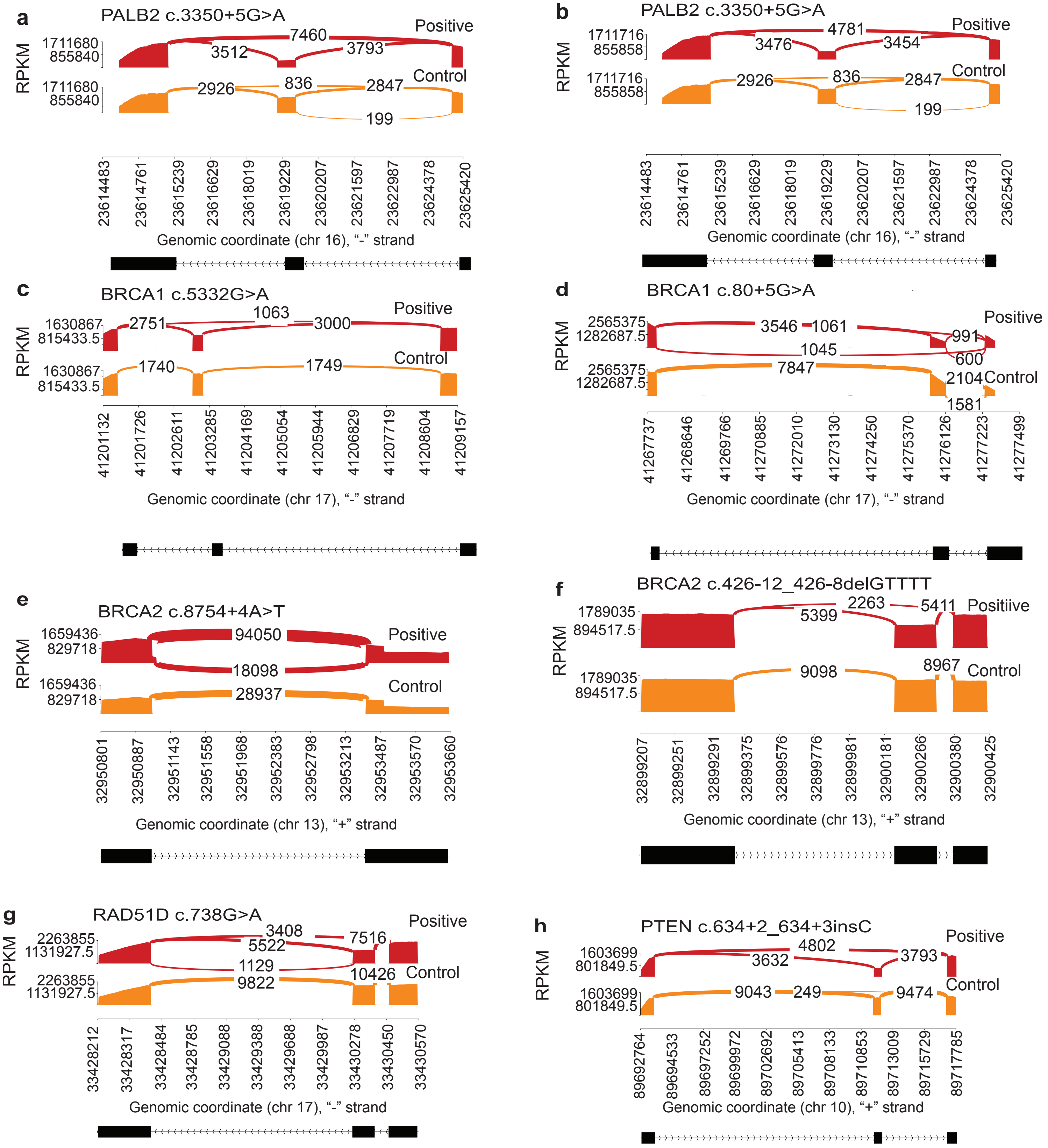

Supplementary Figure 6: Clone-Seq sashimi plots of HBOC pathogenic variants. a-b) *PALB2* r.3202_3350del149 CDS12 full exon skipping c) *BRCA1* r.5278_5332del55 CDS19 full exon skipping d) *BRCA1* r.-25_80del 5'UTR_CDS1 exon skipping e) *BRCA2* r.8754_8755ins8754+1_8754+46 partial intron 21 insertion f) *BRCA2* r.426_475del50 CDS4 full exon skipping g) *RAD51D* r.668_738del71 CDS8 full exon skipping h) *PTEN* r.493_634del142 CDS6 full exon skipping

Supplementary Figure 7

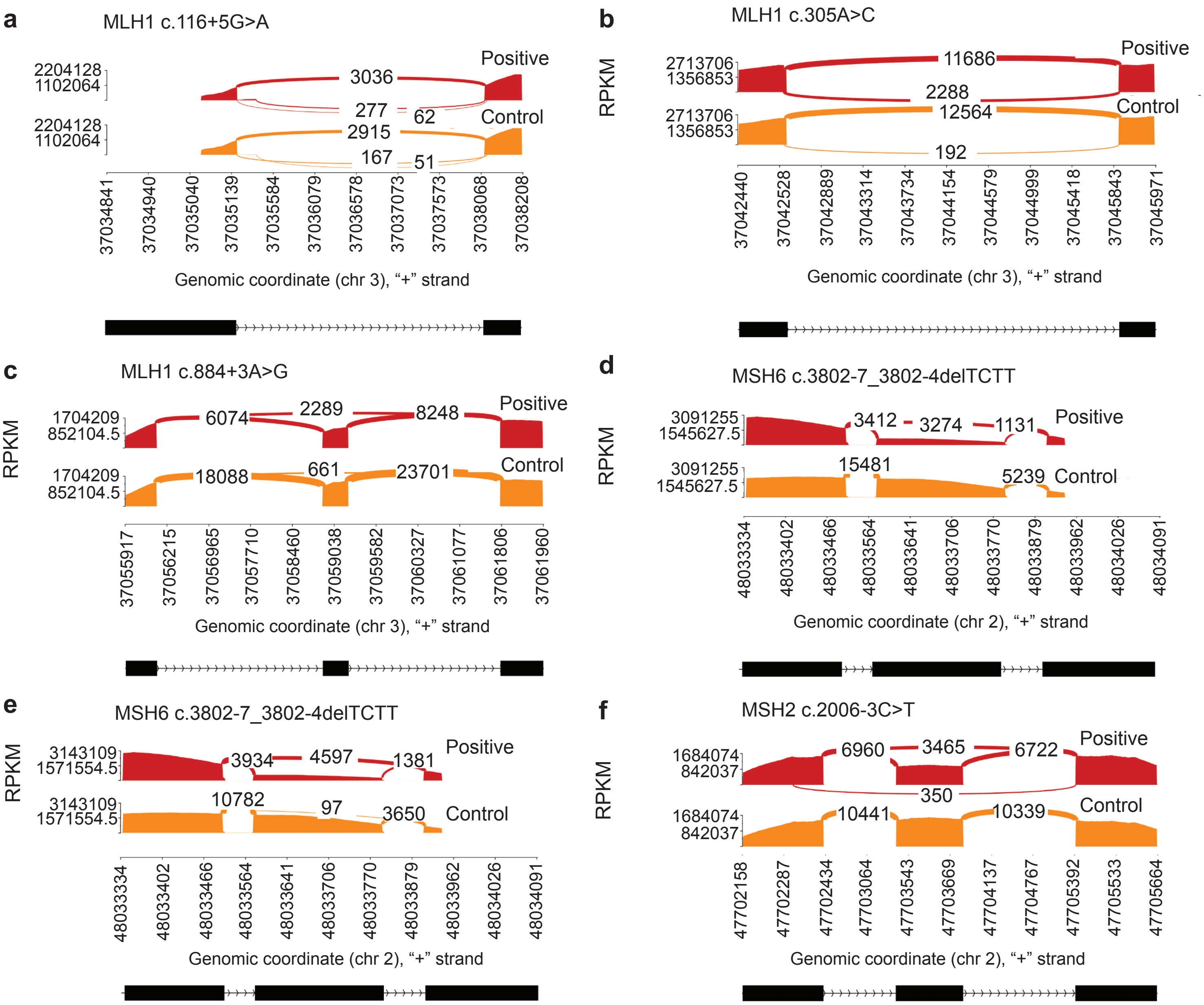

Supplementary Figure 7: Clone-Seq sashimi plots for HNPCC pathogenic variants. a) *MLH1* r.116_117ins116+1_116+227 partial intron 1 retention b) *MLH1* r.302_306del5 CDS3 partial exon skipping c) *MLH1* r.791_884del94 CDS10 full exon skipping d-e) *MSH6* r.3802_4001del200 CDS9 full exon skipping f) *MSH2* r.2006_2210del205 CDS13 full exon skipping

Supplementary Figure 8

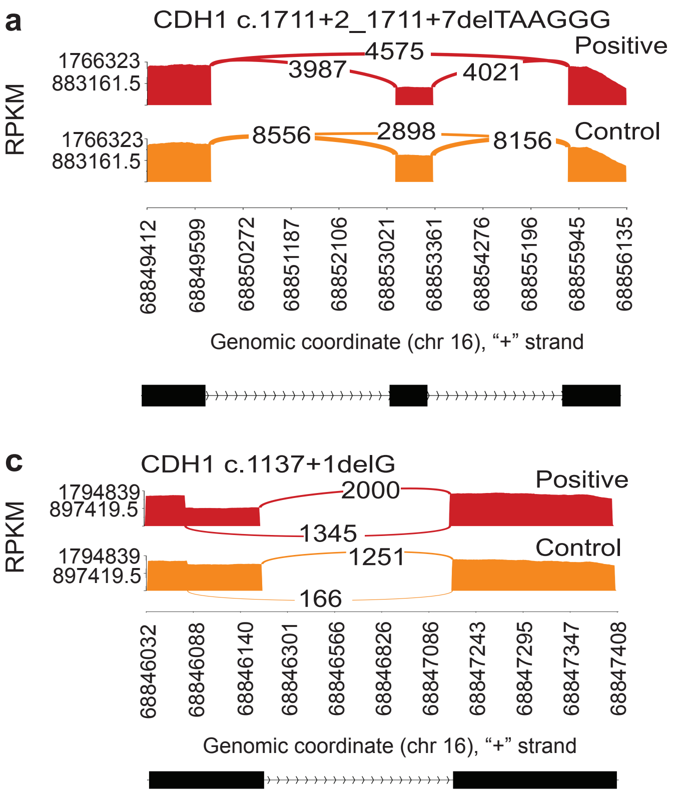

Supplementary Figure 8: Clone-Seq sashimi plots for *CDH1* pathogenic variants. a) r.1566_1711del146 CDS11 full exon skipping b-d) r.1055_1137del83 CDS8 partial exon skipping caused by three distinct germline *CDH1* variants.

Supplementary Figure 9

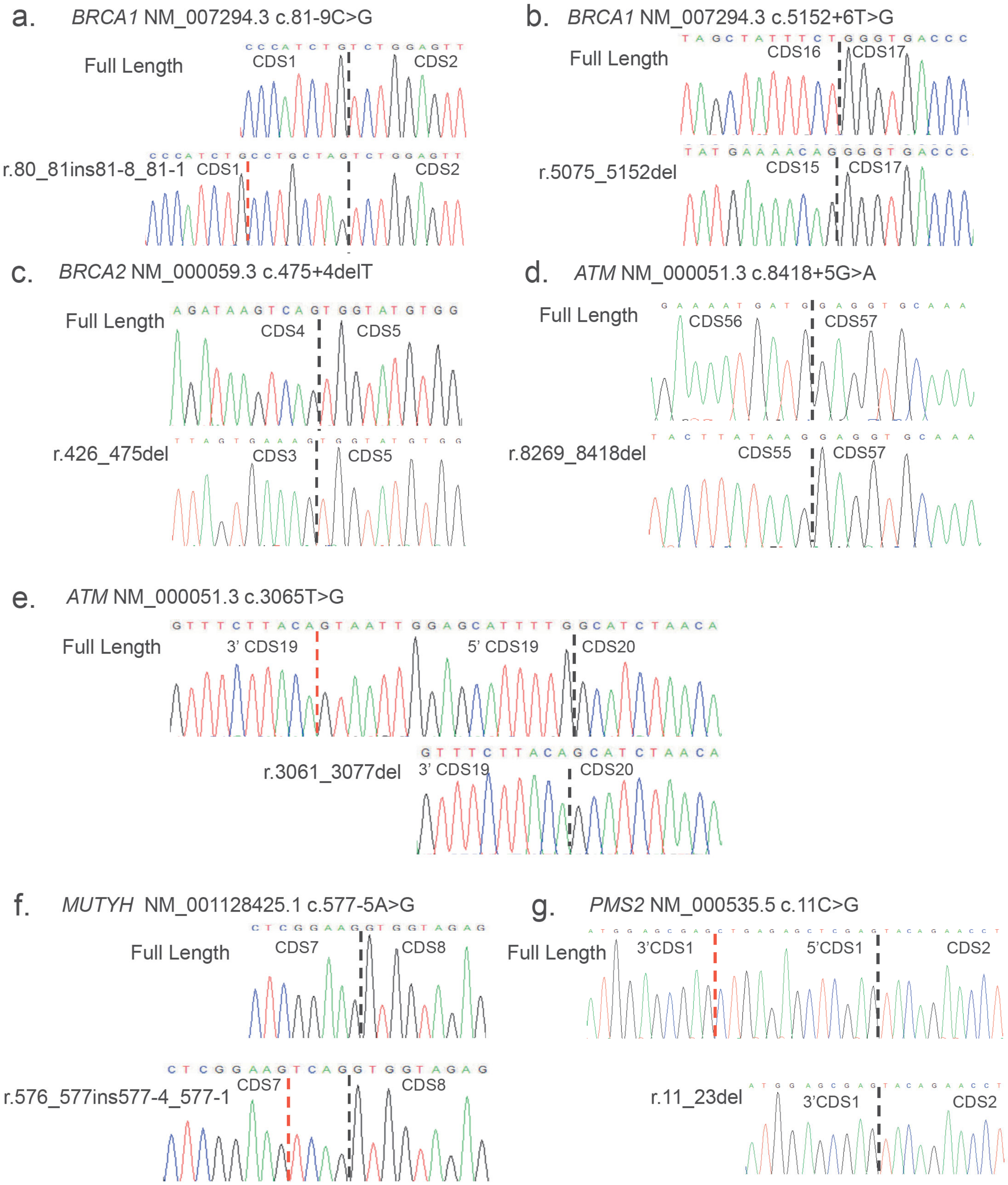

Supplementary Figure 9: Sanger sequencing confirmation of novel variants presented in Figure 4. Individual colonies cloned with RT-PCR products derived from the probands and healthy controls were Sanger sequenced. A. *BRCA1* c.81-9C>G leads to inclusion of 8bp of CDS intron 1 (bottom panel, red line). 66.7% of colonies derived from the proband (n = 15) and 0% of colonies derived from the healthy control (n = 18) were positive for r.80_81ins81-8_81-1; B. *BRCA1* c.5152+6T>G leads to skipping of CDS16 (bottom panel). 52.9% of colonies derived from the proband (n = 19) and 0% of colonies derived from the healthy control (n = 18) were positive for r.5075_5152del; C. *BRCA2* c.475_4delT leads to skipping of CDS4 (bottom panel). 65.0% of colonies derived from the proband (n = 20) and 0% of the colonies derived from the healthy control (n = 14) were positive for r.426_475del. D. *ATM* c.8418+5G>A leads to skipping of CDS56. 70.0% of colonies derived from the proband (n = 20) and 5.0% of the colonies derived from the healthy control (n = 20) were positive for r.8269_8418del; E. *ATM* c.3065T>G leads to a partial deletion of the 5’ end of CDS19. 66.7% of colonies derived from the proband (n = 19) and 0% of the colonies derived from the healthy control (n = 20) were positive for r.3061_3077del; F. *MUTYH* c.577-5A>G leads to an inclusion of 4bp of CDS intron 7. 5.6% of colonies derived from the proband (n = 20) and 0% of the colonies derived from the healthy control (n = 18) were positive for r.576_577ins577-4_577-1; G. *PMS2* c.11C>G leads to a partial deletion of 5’ CDS1. 36.9% of colonies derived from the proband (n = 20) and 0% of the colonies derived from the healthy control (n = 18) were positive for r.11_23del.

Supplementary Table 1: Bait capture RNA-seq vs. GTEx median TPM

Gene	Average bait capture RNA-seq TPM in 345 healthy donors	GTEx median TPM
BRIP1	265.03	0.09
BRCA2	553.08	0.21
CDH1	933.41	0.21
RAD51D	1071.59	1.07
CHEK2	1094.21	1.26
BRCA1	1290.42	0.88
RAD51C	1759.22	0.67
PALB2	2021.01	0.85
PMS2	2523.55	2.08
MUTYH	2650.53	4.09
NF1	2728.18	1.67
MSH2	4966.04	1.36
MLH1	7473.10	6.61
APC	8003.44	1.82
TP53	8458.23	7.70
MSH6	10242.65	2.32
ATM	18465.96	3.50
PTEN	18475.69	40.27

Supplementary Table 2: Comparison of positive control PSI from blood with mean PSI from healthy controls

Gene/c dot variant/r dot variant	Postive Control PSI	Healthy Control Mean PSI (±SD)
APC c.136-1G>A r.136_220del	44.77	1.59±1.28
BRCA1 c.5332G>A r.5278_5332del55	38.41	9.88±10.27
ATM c.73-3C>G r.73_76del4	35.47	0.41±0.48
CDH1 c.1711+2_1711+7delTAAGGG r.1566_1711del	35.32	13.58±11.62
BRCA1 c.5152+5G>T r.5075_5152del	34.08	Absent from controls
CDH1 c.1057G>A r.1055_1137del	33.52	6.03±6.83
BRCA2 c.8754+4A>T r.8754_8755ins8754+1_8754+46	29.32	5.92±5.45
CDH1 c.1137+1delG r.1055_1137del	27.1	6.03±6.83
MLH1 c.305A>C r.302_306del	26.86	4.5±3
CDH1 c.2440-2A>G r.2440_2449del10	25.84	Absent from controls
PALB2 c.2559C>T r.2558_2586del	25.14	Absent from controls
PALB2 c.3350+5G>A r.3202_3350del	22.82	9.23±9.09
MSH6 c.3802-7_3802-4delTCTT r.3802_4001del	22.8	1.99±3.15
MLH1 c.116+5G>A r.116_117ins116+1_116+227	22.68	7.93±5.25
APC c.135+1G>T r.135_136ins135+1_135+69	21.97	1.45±1.64
MSH2 c.1277-14C>G r.1276_1277ins1277-13_1277-1	21.22	Absent from controls
CDH1 c.1137+1delG r.1055_1137del	20.48	6.03±6.83
PALB2 c.3350+5G>A r.3202_3350del	19.97	9.23±9.09
BRCA2 c.426-12_426-8delGTTTT r.426_475del50	19.66	9.82±8.56
BRCA1 c.80+5G>A r.-25_80del	17.61	8.3±10.31
PTEN c.634+2_634+3insC r.493_634del	17.55	1.47±0.69
MSH6 c.3802-7_3802-4delTCTT r.3802_4001del	13.84	1.99±3.15
MSH2 c.1277-14C>G r.1277_1386del	13.5	2.84±3.58
MLH1 c.884+3A>G r.791_884del	12.44	3.02±2.59
PALB2 c.3113+5G>C r.2997_3113del117	10.69	2.53±1.21
MLH1 c.2103+3A>G r.1990_2103del	10.27	Absent from controls
RAD51D c.738G>A r.668_738del	8.31	3.15±0.87
MSH2 c.2006-3T>G r.2006_2210del205	6.94	0.93±0.64
MSH6 c.3417C>T r.3416_3438del	6.31	Absent from controls

Supplementary Table 3: 18 hereditary cancer predisposition

Gene	Isoform
APC	NM_000038.5
APC	NM_001127511.2
ATM	NM_000051.3
BRCA1	NM_007294.3
BRCA2	NM_000059.3
BRIP1	NM_032043.2
CHEK2	NM_007194.3
CDH1	NM_004360.3
MLH1	NM_000249.3
MSH2	NM_000251.1
MSH6	NM_000179.2
PMS2 EX1-10	NM_000535.5
MUTYH	NM_001128425.1
NF1	NM _000267.3
PTEN	NM_000314.4
PALB2	NM_024675.3
RAD51C	NM_058216.1
RAD51D	NM_002878.3
TP53	NM_000546.4
